# Longitudinal associations of changes in physical activity and TV viewing with chronic musculoskeletal pain in Brazilian schoolteachers

**DOI:** 10.1371/journal.pone.0234609

**Published:** 2020-06-17

**Authors:** Mayara Cristina da Silva Santos, Flávia Lopes Gabani, Douglas Fernando Dias, Selma Maffei de Andrade, Alberto Durán González, Mathias Roberto Loch, Arthur Eumann Mesas

**Affiliations:** 1 Department of Public Health, State University of Londrina, Londrina, Parana, Brazil; 2 Universidad de Castilla-La Mancha, Health and Social Research Centre, Cuenca, Spain; University of Ottawa Heart Institute, CANADA

## Abstract

This study analyzed the longitudinal association of changes in leisure-time physical activity (LTPA) practice and television viewing (TV viewing) with chronic musculoskeletal pain (CMP). The data about LTPA, TV viewing, and CMP were obtained in 2012 and after 24 months through individual interviews with schoolteachers from elementary and secondary education public schools in a large city in the southern region of Brazil. The statistical analysis was performed using generalized estimating equation regression models adjusted for sex, age, body mass index and depression. A total of 527 schoolteachers were studied, among which 66.6% were women, and the median age was 42 years (interquartile range: 34 to 49). A total of 170 (32.3%) participants reported CMP at baseline and 130 (24.7%) at follow-up. Both LTPA and TV viewing were independently and significantly associated with CMP regardless of all adjustment variables. Concretely, increasing LTPA by 60 minutes/week was associated with a 6.2% lower likelihood of CMP, and increasing TV viewing by 30 minutes/day was associated with a 5.1% higher likelihood of having CMP among the participants. In summary, this study showed that LTPA and TV viewing have independent and opposite relationships with the longitudinal risk of CMP, which suggests that the potential benefits obtained from practicing more LTPA are insufficient to compensate for the potential detrimental effect of viewing TV for longer with respect to the CMP.

## Introduction

Chronic musculoskeletal pain (CMP) is a public health problem present in most countries of the world and has a direct impact on health services [1]. In primary care, for example, chronic pain is one of the main reasons for seeking care [2]. The body regions commonly affected by CMP are the back [3,4] and upper and lower limbs [5,6]. Some health conditions often associated with CMP are tension, fatigue [7], depression and low quality of life scores [8,9].

Some of the risk factors for CMP are age, sex, depression, anxiety, sleep disorders and obesity. Among management strategies for pain symptomatology are posture correction, stretching, and patient-centered multidisciplinary team interventions. In addition, the practice of physical activity and the reduction of sedentary behaviors are recommended nonpharmacological strategies for the prevention and control of CMP [10,11]. In a recent meta-analysis of prospective studies, the practice of leisure-time physical activity (LTPA) was associated with a 16.0% reduction in the risk of chronic low back pain when comparing individuals with regular physical activity (3 to 4 times a week, totaling between 2 and 4 hours per week) with individuals who did not practice physical activity regularly [12]. Another meta-analysis of studies with adults indicated that increasing weekly physical activity frequency has a chronic pain reduction effect, although the authors concluded that available evidence is not sufficient to identify dose effects of physical activity in relation to analgesia [13]. In addition, there is evidence that reducing sitting time during the day may also lead to beneficial effects on chronic pain control in specific regions, such as the lower back [14].

There is evidence that the practice of physical activity and the reduction of television viewing (TV viewing) are associated with the improvement of CMP symptoms [12,15]. However, it is unclear to what extent changes in these behaviors may influence pain in specific population groups, such as schoolteachers, among whom CMP is highly prevalent [10,15,16]. Previous cross-sectional analyses with Brazilian teachers identified a significant association between a higher frequency of physical inactivity and chronic pain [17] and the highest chance of pain in TV watchers for more than an hour daily [15]. In another cross-sectional study with Chinese teachers, the practice of physical exercise for seven hours or more per week was associated with a lower prevalence of musculoskeletal pain [10].

Studies known in the literature on chronic pain, physical activity and TV viewing in teachers are cross-sectional. This relationship needs to be explored by other designs to better understand the relation between behavioral changes and the incidence or persistence of CMP. Therefore, this study aimed to analyze the longitudinal association of changes in LTPA and TV viewing with CMP in elementary and secondary education schoolteachers.

## Materials and methods

### Study design, population and location

This study comprises longitudinal analyses performed with elementary and secondary schoolteachers as a part of the Pro-Mestre study conducted between 2012 and 2014 with the objective of analyzing health, lifestyle and working aspects in this population [18]. In brief, the study included a census of teachers from the 20 largest elementary and secondary public schools (i.e., those with more than 70 teachers) in Londrina, a large city in southern Brazil. Data were collected through self-administered questionnaires and personal interviews conducted at schools by trained undergraduate and graduate students.

The inclusion criteria were as follows: teaching in a classroom for at least one day per week; being responsible for one or more subjects; not being on leave during the research data collection, which lasted three weeks in each school, or until 30 days after the end of it, with new attempts to find teachers who still remained on leave (15 and 30 days after data collection had been concluded). At baseline (2012), among the 1126 teachers who met the inclusion criteria, 63 (5.6%) refused to participate, 65 were on leave (5.8%), and 20 (1.7%) were not located after five contact attempts on different days and times. Thus, the final analysis at baseline included 978 (86.9%) schoolteachers. After 24 months (2014), the self-administered questionnaires and interviews were repeated. At baseline, two teachers refused to be followed. During follow-up interviews, 366 individuals were lost due to an important labor strike among these professionals, and 19 were rescheduled, but the interviews were unscheduled because of the strike. Of the 591 remaining participants, 20 (3.4%) refused to participate, and 40 (6.8%) were not located, resulting in 531 participants. Among the 531 teachers, two had no information about physical activity at follow-up, one did not report their weight, and another did not respond to the question about chronic pain at baseline, resulting in 527 schoolteachers (53.9%)–357 without baseline pain and 170 with baseline pain—who were ultimately analyzed in this study. The baseline characteristics of the final studied sample were very similar to those observed for the original sample (S1 Table).

This project was approved by the local Human Research Ethics Committee at State University of Londrina (protocol numbers: 01817412.9.0000.5231 and 33857114.4.0000.5231). All participants were informed about the study goals, received assurance of their anonymity, and signed a consent form.

### Study variables

#### Chronic musculoskeletal pain (CMP)

The information on chronic pain was obtained with the following questions: 1) “Do you suffer from any type of chronic pain that has affected you for 6 months or more?”, subsequently categorized as yes and no, and 2) “Please point to the region of the body where you feel this pain.” For this last question, the teacher observed a figure of the human body and indicated the affected(s) region(s); more than one answer was allowed. CMP was defined as reported pain in one or more of the following body sites: shoulders, arms, back, knees, legs and feet.

According to the International Association of Study of Pain, a pain symptom is characterized by “an unpleasant sensory and emotional experience associated with actual or potential tissue damage or described in terms of such damage” [19]. Although CMP was assessed with subjective questions in this study, the IASP reinforces the individual’s self-perception of this phenomenon, since even in the absence of quantitative measurement parameters, the existence of pain is real and should be considered [19].

#### Leisure-time physical activity (LTPA)

Information about leisure-time physical activity was obtained with the following questions: 1) “In a normal week (typical), do you do some type of physical activity in the free time at least once a week?” (categorized as yes and no); if the answer was yes, then we asked 2) “How many times a week do you practice physical activity (days)?” (from one to seven times) and 3) “How much time per day do you practice physical activity?” (answered in minutes).

#### TV viewing

The self-reported time (in minutes per day) watching TV during a normal week was used as an estimation of the time spent on sedentary behavior. The average daily time viewing TV was calculated by adding the time reported for weekdays multiplied by five with that reported for weekends multiplied by two and dividing the result by seven. When considering possible markers of sedentary behavior related to cardiometabolic risk factors, self-report that includes TV viewing showed more consistent results when compared to non-TV leisure-time sitting and accelerometry-measured sedentary behavior [20]. A previous study with the same population showed a cross-sectional relationship between TV viewing and CMP [15]. Other authors observed that the substitution of light walking for watching TV was associated with an increased risk of depression in workers [21].

#### Adjustment variables

Data were also obtained for variables considered as potential confounders according to epidemiological importance for the analysis of LTPA and TV viewing with CMP: sex (female and male); age (in years); body mass index (in kg/m^2^); and medical diagnosis of depression reported at baseline (yes and no). Time spent viewing TV was also treated as a covariate when leisure-time physical activity was the main independent variable, and leisure-time physical activity was included as a covariate when TV-viewing was the main independent variable.

### Statistical analysis

Initially, the Shapiro-Wilk normality test was applied to explore continuous variables (age, BMI, LTPA and TV viewing). As none of them fit the normal distribution, they were described with median and interquartile range (IQR), and the nonparametric Wilcoxon sum-rank test was applied to compare baseline and follow-up values. For the binomial variables CMP and depression, the McNemar test was used to compare baseline and follow-up frequencies.

To analyze the association between changes in leisure-time physical activity and TV viewing (independent variables) and CMP (dependent variable), generalized estimating equation (GEE) regression models were used to estimate the odds ratios and their respective 95% confidence intervals. This method is able to account for specificities of the longitudinal design used, especially in view of the large number of losses in the follow-up. It allows analysis of potentially correlated data, since individuals were interviewed repeatedly over time (baseline and follow-up) and, therefore, considers information on the within-participant variability regarding variables that could change over time (e.g., BMI, LTPA and TV viewing and depression). As the unit of time spent with LTPA and TV viewing is one minute, a recoding was proposed at intervals of 60 minutes/week for LTPA and 30 minutes/day for TV viewing so that the interpretation of the odds ratios could be more intuitive. Both main independent variables (LTPA and TV viewing) were simultaneously included in a single model adjusted by sex and age. Then, a second model was built by adding the BMI variable to the previous model. Finally, a last model included the two main independent variables (LTPA and TV viewing), the previous adjustment variables (sex, age and BMI) and the variable for adjustment for depression. This cumulative adjustment was planned, as changes in BMI could have impacts on both CMP [22] and movement-related behaviors [23], and depression may have intensified painful experience and disability during follow-up [24].

Statistical analyses were performed using the command *xtgee* of STATA SE software, version 15 (StataCorp, College Station, TX, USA). For all analyses, the level of statistical significance was set at p<0.05.

## Results

The study population was predominantly female (66.6%), with ages ranging from 19 to 67 years at baseline. There was no significant change in BMI (p-value = 0.16), TV viewing (p-value = 0.64) or depression frequency (p-value = 0.91) from baseline to follow-up. However, a significant increase of almost 30 minutes per week was observed for the time spent on LTPA over time (p-value = 0.008). Moreover, the frequency of CMP decreased from 32.3% to 24.7% (p-value = 0.001) (Table 1).

**Table 1:** Characteristics of the schoolteachers at baseline and follow-up, Londrina/PR, Brazil, 2012–2014. (N = 527).

Variables	Baseline	Follow-up	p-value^a^
Age (years), median (IQR)	42 (34, 49)	44 (36, 51)	<0.001
BMI (kg/m^2^), median (IQR)	25.2 (22.8, 28.2)	25.6 (23.2, 28.6)	0.26
LTPA (minutes/week), median (IQR)	0 (0, 180)	90 (0, 210)	0.008
TV-viewing (minutes/day)^b^, median (IQR)	77 (43, 120)	77 (43, 137)	0.64
Depression, %	15.6	15.4	0.91
Chronic musculoskeletal pain, %	32.3	24.7	0.001

**BMI**: body mass index; **IQR**: interquartile range; **LTPA**: leisure-time physical activity; **TV**: television.

^a^ Obtained with the Wilcoxon sum-rank test (LTPA and TV-viewing variables) or with the McNemar test (binary variables).

^b^ Information on TV viewing was missing for 2 participants at baseline and for 1 participant at follow-up.

The relationship between change in leisure-time physical activity and TV-viewing time and CMP is shown in Table 2. Both LTPA and TV viewing were independently and significantly associated with CMP regardless of sex, age, BMI and depression. Concretely, increasing LTPA by 60 minutes/week was associated with a 6.2% lower likelihood of CMP, and increasing TV viewing by 30 minutes/day was associated with a 5.1% higher likelihood of having CMP among the schoolteachers studied. All adjustment variables were associated with CMP (p<0.05).

**Table 2:** Association between change in leisure-time physical activity behavior and TV viewing (independent variables) and chronic musculoskeletal pain (dependent variable) after 24 months in schoolteachers, Londrina/PR, Brazil, 2012–2014. (N = 527).

Variables	Model^a^ adjusted for sex and age	Previous model plus adjusted for BMI	Previous model plus adjusted for depression
Change in LTPA time (60 minutes/week)^b^	0.938 (0.886, 0.992)*	0.938 (0.886, 0.992)*	0.938 (0.887, 0.993)*
Change in TV-viewing time (30 minutes/day)^b^	1.054 (1.005, 1.106)*	1.054 (1.005, 1.106)*	1.051 (1.001, 1.102)*
Sex (female/male)	1.575 (1.108, 2.239)*	1.575 (1.108, 2.239)*	1.498 (1.055, 2.127)*
Age (y)	1.025 (1.008, 1.041)*	1.025 (1.008, 1.041)*	1.024 (1.007, 1.041)*
Change in BMI (kg/m^2^)		1.038 (1.003, 1.075)*	1.036 (1.002, 1.072)*
Change in depression status (yes/no)			1.873 (1.272, 2.757)*
*Constant*	0.022 (0.006, 0.085)*	0.022 (0.006, 0.085)*	0.024 (0.006, 0.091)*
*Wald Chi2*, *Prob >chi2*	26.09, <0.001	29.92, <0.001	36.19, <0.001

*p-value <0.05

^a^ Values are the odds ratios (95% confidence interval) estimated though the generalized estimating equation (GEE) regression model (parameters used: family: binomial; link: logit; correlation: independent; variance estimators: robust).

^b^ Both variables were simultaneously included in the same model.

## Discussion

This study identified that, if, on the one hand, increasing the time dedicated to leisure-time physical activity (LTPA) may reduce the likelihood of chronic musculoskeletal pain (CMP), increasing the time viewing TV is a change in behavior with potential negative effects for the persistence or incidence of this type of chronic pain. Furthermore, these associations are not interdependent, which suggests that the potential benefits obtained from practicing more LTPA are insufficient to compensate for the potential detrimental effect of viewing TV for longer with respect to the CMP.

The present study expands the set of scientific evidence about the possible benefits of the practice of leisure-time physical activity, since increasing this behavior by 60 minutes per week may prospectively reduce the risk of CMP. In a cross-sectional study with teachers from Ethiopia, the practice of physical activity was also associated with the reduction of chronic low back pain [25]. Moreover, this was also observed in a meta-analysis performed with studies about physical activity, exercise and pain that showed that increasing weekly physical activity decreased the symptoms of chronic pain [13], including persistent pain in the low back region [26]. Regardless of the adoption of sedentary behavior and the presence of depression, the benefits of physical activity could be observed in this study, which is in agreement with the literature [27].

Interestingly, increasing TV-viewing time (a proxy for total sedentary behavior [20] by 30 minutes per day was associated with a greater likelihood of CMP independent of changes in leisure-time physical activity, BMI and depression. The increase in the practice of physical activity according to international recommendations [28] does not guarantee that people will reduce their sedentary behavior. A literature review with occupational groups found that adults spend approximately 60.0% of their time on sedentary behavior and approximately 4.0% of the day involved in moderate physical activity [29]. Thus, specific population groups can have their health compromised if they spend too much time in sedentary activities, even if they reach recommended levels of physical activity [30].

Regarding the association between physical activity and CMP, experimental studies with animals and humans indicate that the practice of regular exercise promotes pain relief by reducing the expression of the serotonin transporter, increasing the level of this neurotransmitter in the synaptic cleft and reducing secondary hyperalgesia by increasing central inhibitory opioids [31,32]. That is, the practice of regular physical activity uses the endogenous inhibitory system for pain reduction. In this sense, moderate and vigorous physical activity and according to the characteristics socioeconomic and health conditions of each individual can be considered a nonpharmacological alternative to control pain symptoms [27].

Some methodological considerations must be addressed for the correct interpretation of these results. First, the teachers’ strike that occurred during the follow-up data collection caused many losses among the study population and limited the statistical power. However, this event possibly had a minor effect on the study results, as the main characteristics of the original population remained very similar among those teachers finally studied. Second, information on physical activity and TV viewing time was obtained through the interviewee’s self-report, and thus, the accuracy of the results may be affected by recall and information bias. In addition, even though the information on physical activity was not obtained with a validated questionnaire, they were based on questions that allowed us to characterize the interviewee’s report as to the type, frequency and duration of physical activities performed during free time in a usual week, which enabled the analyses proposed. Moreover, although questionnaires are commonly adopted in epidemiological studies [33], accelerometry could provide more accurate measurements of physical activity [34]. Third, the longitudinal design is a strength of this study, and the analysis strategy used (GEE) allowed us to explore the effects of physical activity and TV viewing on CMP considering changes that occurred over the period for these and for the adjustment variables, controlling for the correlation between the responses of teachers at two different times and mitigating the effect of missing values [35]. Lastly, as no data were collected on the use of analgesics, it was not possible to examine the effects of the drugs used to control pain. Thus, it cannot be said that the lower likelihood of presenting CMP at follow-up is due exclusively to the practice of physical activity. Moreover, it was not possible to examine the effect of pain intensity on the performance of physical activity and TV viewing.

In summary, this study showed that leisure-time physical activity is a behavior potentially involved in beneficial health outcomes such as chronic musculoskeletal pain symptoms. Longitudinal studies based on objective measures, intensity and other domains of physical activity are still needed to examine a possible dose-response effect. Although our results come from a study with a longitudinal observational design, they are in the same direction as experimental [13] studies that provide relevant evidence that the encouragement to walk or cycle to and from work and the implementation of physical activity promotion in the work environment can play a relevant role in workers’ health and quality of life. Together with an adequate supply of health services for the diagnosis, treatment and control of painful conditions, the promotion of physical activity among schoolteachers is a strategy with the potential to contribute to the reduction of absenteeism and presenteeism resulting from CMP, benefiting not only the worker but also the educational system.

## Supporting information

S1 Table Characteristics of the schoolteachers in the total original sample and in the final studied sample, Londrina/PR, Brazil, 2012–2014. (DOCX)
