# Supplementary material for: Longitudinal associations of changes in physical activity and TV viewing with chronic musculoskeletal pain in Brazilian schoolteachers
Source: PLoS One. 2020 Jun 17;15(6):e0234609. doi: 10.1371/journal.pone.0234609 (PMC7299367; doi:10.1371/journal.pone.0234609)
Supplement: S1 Table — (DOCX) [file pone.0234609.s001.docx]

**Table S1.** **Characteristics of the schoolteachers in the total original sample and in the final studied sample, Londrina/PR, Brazil, 2012-2014.**

| **Variables** | **Original study sample** | **Final studied sample** |
| --- | --- | --- |
| Total, n (%) | 978 (100.0) | 527 (53.9) |
| Female sex, n (%) | 670 (68.5) | 351 (66.6) |
| Age (years), median (IQR) | 42 (33, 49) | 42 (34, 49) |
| BMI (kg/m^2^), median (IQR) | 25.3 (22.7, 28.1) | 25.2 (22.8, 28.2) |
| LTPA (min/week), median (IQR) | 0 (0, 180) | 0 (0, 180) |
| TV-viewing (min/day)^a^, median (IQR) | 77 (43, 126) | 77 (43, 120) |
| Depression, % | 152 (15.5) | 82 (15.6) |
| Chronic musculoskeletal pain, % | 299 (30.6) | 170 (32.3) |

**BMI**: body mass index; **IQR**: interquartile range; **LTPA**: leisure-time physical activity; **TV**: television.

^a^ Information on TV viewing was missing for 3 participants of the original sample and for 2 participants of the final studied sample.
